# Supplementary material for: HIVprotI: an integrated web based platform for prediction and design of HIV proteins inhibitors
Source: J Cheminform. 2018 Mar 9;10:12. doi: 10.1186/s13321-018-0266-y (PMC5845081; doi:10.1186/s13321-018-0266-y)
Supplement: Supplementary file 2 — Additional file 2. Source code of HIVProtI web server. [file 13321_2018_266_MOESM2_ESM.zip › HIVprotI_Source-code/hivprotI/weka-3-6-6/doc/weka/classifiers/bayes/net/search/fixed/FromFile.html]

FromFile


---


|  |  |  |
| --- | --- | --- |
| **Overview** \| **Package** \| **Class** \| **Tree** \| **Deprecated** \| **Index** \| **Help** \| **Weka's home** | |  |
| PREV CLASS   **NEXT CLASS** | **FRAMES**    **NO FRAMES**     **All Classes** |  |
| SUMMARY: NESTED \| FIELD \| CONSTR \| METHOD | DETAIL: FIELD \| CONSTR \| METHOD |  |


---


## weka.classifiers.bayes.net.search.fixed Class FromFile

```
java.lang.Object
  weka.classifiers.bayes.net.search.SearchAlgorithm
      weka.classifiers.bayes.net.search.fixed.FromFile
```

**All Implemented Interfaces:**: java.io.Serializable, OptionHandler, RevisionHandler

---

``` public class FromFile extends SearchAlgorithm ```

The FromFile reads the structure of a Bayes net from a file in BIFF format.


Valid options are:

```
 -B <BIF File>
  Name of file containing network structure in BIF format
```

**Version:**
:   $Revision: 1.8 $

**Author:**
:   Remco Bouckaert

**See Also:**: Serialized Form

---

**Constructor Summary**

`FromFile()`


| **Method Summary** | |
| --- | --- |
| `void` | `buildStructure(BayesNet bayesNet, Instances instances)`             buildStructure determines the network structure/graph of the network. |
| `java.lang.String` | `getBIFFile()`             Get name of network in BIF file to read structure from |
| `java.lang.String[]` | `getOptions()`             Gets the current settings of the search algorithm. |
| `java.lang.String` | `getRevision()`             Returns the revision string. |
| `java.lang.String` | `globalInfo()`             Returns a string describing this object |
| `java.util.Enumeration` | `listOptions()`             Returns an enumeration describing the available options. |
| `void` | `setBIFFile(java.lang.String sBIFFile)`             Set name of network in BIF file to read structure from |
| `void` | `setOptions(java.lang.String[] options)`             Parses a given list of options. |

**Methods inherited from class weka.classifiers.bayes.net.search.SearchAlgorithm**

`initAsNaiveBayesTipText, maxNrOfParentsTipText, toString`

**Methods inherited from class java.lang.Object**

`equals, getClass, hashCode, notify, notifyAll, wait, wait, wait`

**Constructor Detail**

### FromFile

```
public FromFile()
```


**Method Detail**

### globalInfo

```
public java.lang.String globalInfo()
```

:   Returns a string describing this object

    :   **Returns:**: a description of the classifier suitable for displaying in the explorer/experimenter gui

---


### buildStructure

```
public void buildStructure(BayesNet bayesNet,
                           Instances instances)
                    throws java.lang.Exception
```

:   **Description copied from class: `SearchAlgorithm`**
:   buildStructure determines the network structure/graph of the network.
    The default behavior is creating a network where all nodes have the first
    node as its parent (i.e., a BayesNet that behaves like a naive Bayes classifier).
    This method can be overridden by derived classes to restrict the class
    of network structures that are acceptable.

    :   **Overrides:**: `buildStructure` in class `SearchAlgorithm`
    :   **Parameters:**: `bayesNet` -: `instances` - the instances to work with **Throws:**: `java.lang.Exception` - if attribute from BIF file could not be found

---


### setBIFFile

```
public void setBIFFile(java.lang.String sBIFFile)
```

:   Set name of network in BIF file to read structure from

    :   **Parameters:**: `sBIFFile` - the name of the BIF file

---


### getBIFFile

```
public java.lang.String getBIFFile()
```

:   Get name of network in BIF file to read structure from

    :   **Returns:**: BIF file name

---


### listOptions

```
public java.util.Enumeration listOptions()
```

:   Returns an enumeration describing the available options.

    :   **Specified by:**: `listOptions` in interface `OptionHandler` **Overrides:**: `listOptions` in class `SearchAlgorithm`
    :   **Returns:**: an enumeration of all the available options.

---


### setOptions

```
public void setOptions(java.lang.String[] options)
                throws java.lang.Exception
```

:   Parses a given list of options.


    Valid options are:

    ```
     -B <BIF File>
      Name of file containing network structure in BIF format
    ```

    :   **Specified by:**: `setOptions` in interface `OptionHandler` **Overrides:**: `setOptions` in class `SearchAlgorithm`
    :   **Parameters:**: `options` - the list of options as an array of strings **Throws:**: `java.lang.Exception` - if an option is not supported

---


### getOptions

```
public java.lang.String[] getOptions()
```

:   Gets the current settings of the search algorithm.

    :   **Specified by:**: `getOptions` in interface `OptionHandler` **Overrides:**: `getOptions` in class `SearchAlgorithm`
    :   **Returns:**: an array of strings suitable for passing to setOptions

---


### getRevision

```
public java.lang.String getRevision()
```

:   Returns the revision string.

    :   **Specified by:**: `getRevision` in interface `RevisionHandler` **Overrides:**: `getRevision` in class `SearchAlgorithm`
    :   **Returns:**: the revision


---


|  |  |  |
| --- | --- | --- |
| **Overview** \| **Package** \| **Class** \| **Tree** \| **Deprecated** \| **Index** \| **Help** \| **Weka's home** | |  |
| PREV CLASS   **NEXT CLASS** | **FRAMES**    **NO FRAMES**     **All Classes** |  |
| SUMMARY: NESTED \| FIELD \| CONSTR \| METHOD | DETAIL: FIELD \| CONSTR \| METHOD |  |


---
